# Septic shock in the prehospital setting: a scoping review

**DOI:** 10.1186/s13049-024-01282-2

**Published:** 2024-11-14

**Authors:** Ricardo Sebastian Pinto-Villalba, Daniela Balseca-Arellano, Jose E. Leon-Rojas

**Affiliations:** 1 Carrera de Atención Prehospitalaria y Emergencias, Facultad de Ciencias de las Salud Eugenio Espejo, Universidad UTE, Quito, Ecuador; 2 Escuela de Medicina, Universidad de las Américas (UDLA), Quito, Ecuador; 3 Health Sciences Research Department, Medignosis, Quito Ecuador; 4 Sociedad Ecuatoriana de Reanimación Cardiopulmonar (SERCA), Quito, Ecuador

**Keywords:** Sepsis, Septic shock, Prehospital, Emergency medical services, Prehospital management

## Abstract

Septic shock (SS) is a potential life-threatening condition in which an early identification and immediate therapy stand out as the main cornerstones to improve survival chance; in this context, emergency medical services (EMS) become key to reduce the time between diagnosis and management in the ICU or emergency department. However, guidelines for the prehospital management of SS patients remains unclear, and literature around this topic is scant. Our scoping review was conducted following the PICO framework and a search strategy related to septic shock management and diagnosis in prehospital settings was executed in PubMed, Scopus and Virtual Health Library; articles in English and Spanish from 2015, onwards, were screened by the authors and selected by mutual consensus. Our aim is to analyze the prehospital management strategies of SS reported in the literature, and to showcase and summarize the screening tools, demographic factors, clinical manifestations and prognostic factors of SS in the prehospital setting.

## Introduction

Septic shock (SS) is a complex medical disorder caused by sepsis, a deadly medical condition that Homer’s poetry referenced and was formally recognized in 1992 [1, 2]. Sepsis and SS pathophysiology is complex and under study; it involves increased vascular permeability, peripheral vasodilation, hypovolemia, and ventricular dysfunction [3–5]. Fluid loss via diarrhea, vomiting, fever, tachypnea, and capillary leakage causes hypovolemia, decreasing cellular perfusion, increasing anaerobic metabolism and lactic acid generation (serum lactate can exceed 2 mmol/L even after appointment fluid resuscitation) [4–6]. This often requires vasopressor therapy to achieve a mean blood pressure of 65 mm Hg or higher [6].

Depending on the infection’s origin and stage, septic shock might produce coughing, dyspnea (if pulmonary), vomiting, and diarrhea (if gastrointestinal); fever, tachycardia, and tachypnea are often reported [5, 7]. Infection alone does not indicate sepsis or SS; the latter is diagnosed by finding systemic hypoperfusion, hypotension, and anaerobic metabolism markers in an infectious illness [1, 7].

Septic shock is common worldwide. Bauer’s meta-analysis found 93,000 global diagnosed cases with an average 30-day mortality rate of 34.7% (95% CI: 32.6–32.9%) and 90-day mortality of 38.5% [8]. With 34% and 68%, respectively, developed and developing nations have different SS mortality rates [8–10]. Time to antibiotic administration and admission are major factors in these disparities, as early detection and treatment improve survival [9–12].

Liu et al. found that 40% of SS patients in the emergency room or intensive care unit are evacuated by emergency medical services (EMS), highlighting the necessity for early detection, and directed therapy to enhance patient outcomes [13]. Experts and the Surviving Sepsis Campaign agree that early and aggressive therapy can boost survival, reduce hospitalization, and reduce complications [14–17]. Sepsis onset, prehospital diagnosis, and ED/ICU admission vary greatly. In a study of 361 community-dwelling sepsis patients, Peltan et al. found that prehospital staff administered antibiotics faster (103 vs. 144 min) than non-EMS personnel [16]. Given the frequency of hospitalizations and the relevance of prehospital support in improving patient outcomes before ED or ICU admission, an evidence-based prehospital management plan is needed; especially when evidence suggests that prehospital management of SS may improve survival rates [15, 18, 19]. Therefore, this scoping review aims to gather and integrate all relevant information on EMS prehospital SS management to establish a comprehensive framework for its field management.

## Methods

This study was conducted following the Preferred Reporting Items for Systematic Reviews and Meta-Analyses Extension for Scooping Reviews (PRISMA-ScR). Due to the scoping nature of this study its protocol has not been prospectively registered.

### Eligibility criteria

Primary and secondary studies, in English or Spanish, dealing with SS in adults in prehospital settings reporting any type of therapy or intervention, screening tool or diagnostic criteria were included. All studies involving pediatric patients (< 18 years old), pregnant women, animals or mannequins were excluded; articles published before 2015 were rejected. However, studies which explain pathophysiology, demography, clinical presentation, diagnostic tools, and other topics related to SS in prehospital settings were included.

### Information sources

Studies were identified in Scopus, PubMed, and Biblioteca Virtual en Salud (BVS); the references of selected articles were also screened. No filters were used. BVS includes scientific literature from Latin America and the Caribbean (i.e., in developing countries).

### Search strategy

Our search strategy was developed following the PICO framework with key terms related to SS management in prehospital settings; the following key terms, with variations, were used: septic shock, prehospital, emergency medical services, screening, diagnosis, therapy, and management. The last search was executed in July 2023.

### Study selection

Two blinded reviewers carried out an independent and standardized evaluation of the selected studies using Mendeley Reference Manager. Deduplication was performed automatically using Mendeley Reference Manager and two stages of screening were performed. During the first stage, reviewers screened the titles and abstracts of articles, while the second stage involved full-text text review.

**Fig. 1:**
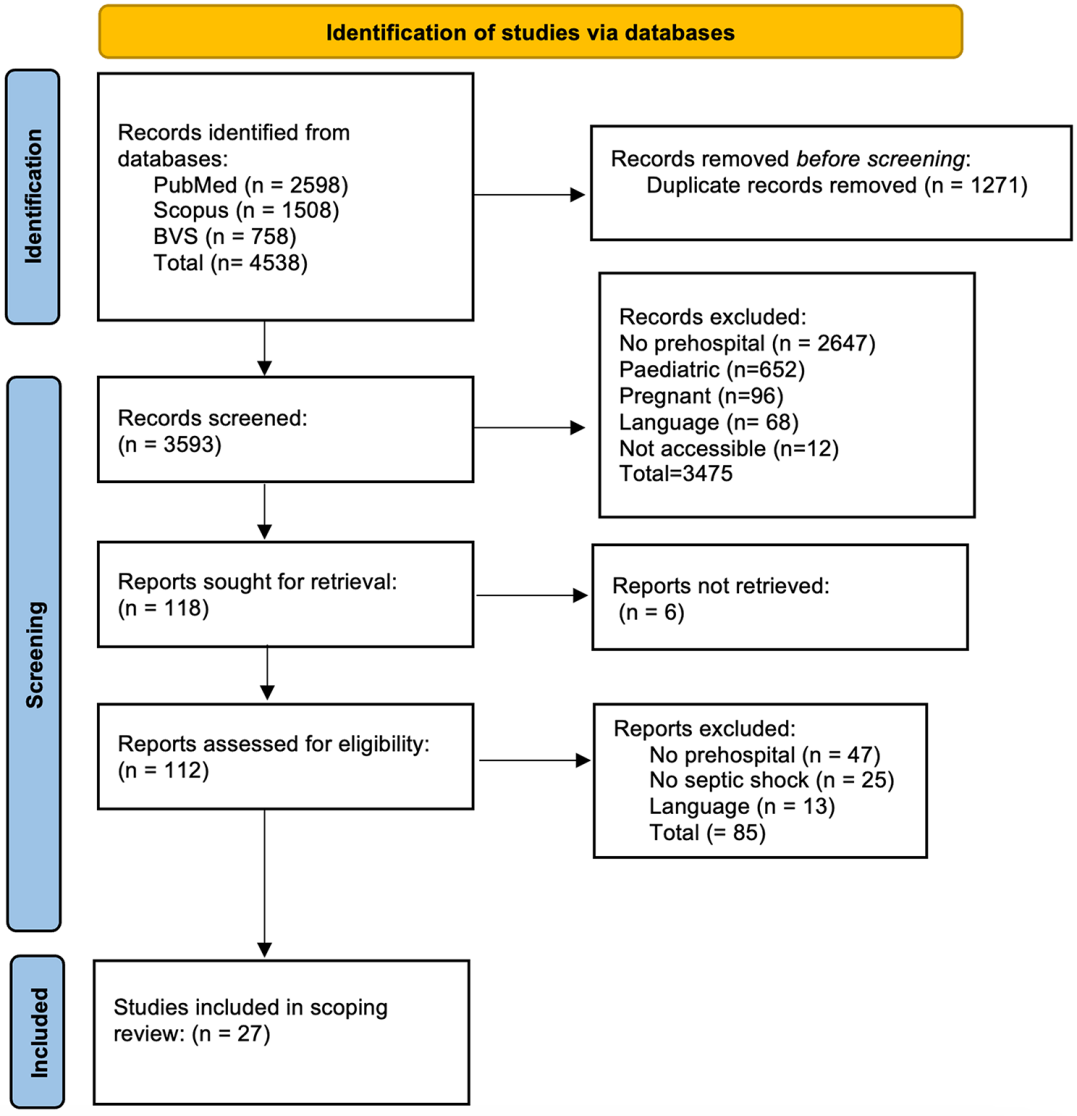
PRISMA-ScR Flowchart

Data synthesis is depicted according to the PRISMA-ScR flow chart (Fig. 1) providing further information regarding the number of studies selected at each stage as well as the reasons for exclusion. Any discrepancies between the reviewers were resolved by discussion and mutual consensus.

### Data extraction and collection process

For data collection we categorize information according to the topics of epidemiology in prehospital settings, clinical manifestations in prehospital settings, screening tools of sepsis and SS, and prehospital management of SS (i.e., oxygenation, fluid therapy, airway management, vasopressor support, antibiotic therapy); to improve clarity and completeness, we have also include a short review on the pathophysiology of SS, as well as the perspectives on future approaches in SS management in the prehospital setting.

## Results

### Literature search

The searches conducted in the databases from 2015 to July 2023 yielded a total of 4538 results, 118 met our inclusion criteria after screening the tittles and abstracts. On full text review, 85 studies were excluded and finally, 27 articles were included (Fig. 1).

### Description of studies

The 27 studies were divided into three themes based on their characteristics (Table 1), namely, epidemiology of SS in prehospital settings (4 studies), diagnosis and screening in prehospital settings (9 studies), and prehospital management of septic shock (14 studies).

**Table 1:** Characteristics of include studies

Nº	Title	Author	Year	Study design	Theme
1	Epidemiology of septic shock in prehospital medical services in five Colombian cities [9]	López-Medina	2020	Cross-sectional	Epidemiology
2	Sepsis and septic shock in emergency departments of Mexico: a multicenter point prevalence study [10]	Gorordo-Delsol LA	2021	Cross-sectional	Epidemiology
3	Emergency Medical Services Care and Sepsis Trajectories [13]	Liu R	2020	Cross-sectional	Epidemiology
4	Time to Treatment and Mortality during Mandated Emergency Care for Sepsis [21]	Seymour CW	2017	Cross-sectional	Epidemiology
5	qSOFA Has Poor Sensitivity for Prehospital Identification of Severe Sepsis and Septic Shock [11]	Dorsett M	2017	Cross-sectional	Diagnosis and screening
6	The prehospital SIGARC score to assess septic shock in-hospital, 30-day and 90-day mortality [12]	Jouffroy R	2021	Cross-sectional	Diagnosis and screening
7	An Early Warning Scoring System to Identify Septic Patients in the Prehospital Setting: The PRESEP Score [25]	Bayer O	2015	Cross-sectional	Diagnosis and screening
8	Prehospital triage of septic patients at the SAMU regulation: Comparison of qSOFA, MRST, MEWS and PRESEP scores [26]	Jouffroy R	2018	Cross-sectional	Diagnosis and screening
9	Identification of adult septic patients in the prehospital setting [27]	Wallgren UM	2015	Cross-sectional	Diagnosis and screening
10	A pilot study exploring the accuracy of pre-hospital sepsis recognition in the North East Ambulance Service [28]	McClelland G	2015	Cross-sectional	Diagnosis and screening
11	Persistently elevated early warning scores and lactate identifies patients at high risk of mortality in suspected sepsis [29]	Hargreaves DS	2015	Cohort Study	Diagnosis and screening
12	A prehospital screening tool utilizing end-tidal carbon dioxide predicts sepsis and severe sepsis [30]	Hunter CL	2016	Cohort Study	Diagnosis and screening
13	Sepsis alerts in EMS and the results of pre-hospital ETCO2 [31]	Weiss SJ	2019	Cohort Study	Diagnosis and screening
14	Pre-hospital mechanical ventilation in septic shock patients [15]	Jouffroy R	2019	Cohort Study	Management
15	Prehospital Care and Emergency Department Door-to-Antibiotic Time in Sepsis [16]	Peltan ID	2018	Cohort Study	Management
16	Impact of Prehospital Antibiotic Therapy on Septic Shock Mortality [17]	Jouffroy R	2021	Cohort Study	Management
17	Prehospital hemodynamic optimisation is associated with a 30-day mortality decrease in patients with septic shock [22]	Jouffroy R	2021	Cohort Study	Management
18	Prognosis value of partial arterial oxygen pressure in patients with septic shock subjected to pre-hospital invasive ventilation [32]	Jouffroy R	2019	Cross-sectional	Management
19	The role of point of care ultrasound in prehospital critical care: a systematic review [45]	Bøtker MT	2018	Systematic Review	Management
20	Ultrasound in Sepsis and Septic Shock—From Diagnosis to Treatment [46]	Tullo G	2023	Review	Management
21	Fluid resuscitation in pre-hospital management of septic shock [47]	Jouffroy R	2018	Cross-sectional	Management
22	Adverse events associated with administration of vasopressor medications through a peripheral intravenous catheter [49]	Owen VS	2021	Systematic review and Meta-analysis	Management
23	Prehospital norepinephrine administration reduces 30-day mortality among septic shock patients [51]	Jouffroy R	2022	Cohort Study	Management
24	High-dose vasoactive agents in aeromedical retrievals for septic shock: A role for vasopressin? [53]	Aston B	2023	Cross-sectional	Management
25	Time-to-antibiotics and clinical outcomes in patients with sepsis and septic shock: a prospective nationwide multicenter cohort study [53]	Im Y	2022	Cohort Study	Management
26	Prehospital administration of broad-spectrum antibiotics for sepsis patients: A systematic review and meta-analysis [56]	Varney J	2022	Systematic review and Meta-analysis	Management
27	Paramedic-Initiated CMS Sepsis Core Measure Bundle Prior to Hospital Arrival: A Stepwise Approach [57]	Walchok JG	2017	Cohort Study	Management

#### Epidemiological profile of septic shock in prehospital settings

SS affects hospitals worldwide, with 34–68% mortality in affluent and poor nations [8–10]. Several variables explain these mortality rate discrepancies. However, a vigorous prehospital therapeutic strategy enhances survival to 68.9% (95%CI, 68.4–69.4%) [14, 20, 21]. Early diagnosis and proactive management by EMS are critical.

Prehospital SS is more common in elderly males (median age 64 years), accounting for 56.9% of cases [10]. Preclinical evidence suggests estrogen’s protection may help females survive infection, while testosterone immunosuppression may have a role in sex- and gender-related SS [10]. In the prehospital situation, SS is related with arterial hypertension (22–38%), cancer (19–29%), diabetes (12–22%), heart failure (8–12%), and COPD (5–17%) [9]. Infection origins are mostly lung (64%), gastrointestinal (20%), and genitourinary (14%), which favor gram-negative bacteria (62%), followed by gram-positive bacteria (47%), and fungi (19%) [9].

#### Clinical manifestations in prehospital settings

The primary clinical manifestations observed in the prehospital setting, in addition to signs and symptoms specific to an infection in any system, included hypotension (systolic blood pressure < 100 mmHg, diastolic blood pressure < 58 mmHg, and/or mean arterial pressure (MAP) < 70 mmHg in the presence of vasopressors and fluids; or systolic blood pressure < 92 mmHg, diastolic blood pressure < 54 mmHg, and/or MAP < 66 mmHg without vasopressors or fluids); tachycardia (> 111 beats per minute), tachypnea (> 28 breaths per minute), oxygen saturation < 88%, body temperature > 38.5 °C, Glasgow Coma Scale (GCS) score < 14, skin mottling score (SMS) > 2, capillary refill time (CRT) > 7 s, and glycemia > 166 mg/dL [20, 22]. Additionally, data suggests that patients with higher values in blood pressure, oxygen saturation, GCS score, body temperature, and glycemia, as well as lower values in heart rate, respiratory rate, SMS, and CRT at the time of EMS arrival have a higher chance of survival [20, 22]. However, these manifestations may vary widely depending on the stage of the disease, time to recognition, and initial prehospital management.

#### Screening tools of sepsis and septic shock

EMS is often the first point of contact between patients and healthcare experts, therefore accurate sepsis and SS diagnosis is critical. EMS must have accurate screening tools for this. However, prehospital sepsis and SS diagnosis remains difficult. Numerous screening tools have been created for prehospital environments, however their sensitivity and specificity vary, resulting in an ongoing debate about the optimal instrument. Definitions are a major hurdle to creating a screening tool. Despite SEPSIS-3 recommendations, numerous writers use “severe sepsis” (SEPSIS-2 language) and the Société Française d’Anesthésie et de Réanimation/Société de Réanimation de Langue Française (SFAR-SRLF) criteria [6, 23, 24].

Systemic Inflammatory Response Syndrome (SIRS), quick Sequential Organ Failure Assessment (qSOFA), Prehospital Early Sepsis Detection (PRESEP) (Table 2), Modified Early Warning Score (MEWS), Modified Robson screening tool (mRST) (Table 3), National Early Warning Score (NEWS), and SIGARC score (Table 4) are the main prehospital screening tools [11, 12, 14, 25–28].

**Table 2:** Prehospital Early Sepsis Detection (PRESEP)

Parameter	Value
Temperature > 38 °C	4
Temperature < 36 °C	1
Heart rate > 90 beats/min	2
Respiratory rate > 22 breaths/min	1
SaO2 < 92%	2
Systolic blood pressure < 90 mm Hg	2

Note The cut off value for a possible septic disease ≥ 4 [24]

**Table 3:** Modified robson screening tool (mRST)

Parameter	Value
Temperature > 38,3 °C	1
Temperature < 36 °C	1
Heart rate > 90 beats/min	1
Respiratory rate > 20 breaths/min	1
Altered mental state presented by GCS < 15	1
Blood glucose > 119 mg/dL	1

Note Threshold ≥ 2 to diagnose sepsis. Glasgow come scale (GCS) [25]

**Table 4:** SIGARC score

Parameter	Value
Shock index ≥ 1	1
Glasgow coma scale < 13	1
Age > 65	1
Respiratory rate > 22	1
Comorbidity	1

Note Threshold ≥ 2 to diagnose SS. Comorbidity is defined by the presence of at least 2 underlying conditions: hypertension, coronary heart disease, chronic heart failure, chronic renal failure, chronic obstructive pulmonary disease, diabetes mellitus, history of cancer and human immunodeficiency virus infection [9]

The screening tools SIRS and qSOFA have been criticized for their low specificity and sensitivity in prehospital settings; SIRS has a reported sensitivity of 84.4% and specificity of 39.5%, while qSOFA has a sensitivity of 16.3% and specificity of 97.3% [11]. Additionally, the Surviving Sepsis Campaign has advised against the use of qSOFA [14].

The PRESEP tool (Table 2) was initially introduced as a screening tool with a focus on high precision for prehospital settings; it was reported to have a sensitivity of 85%, specificity of 86%, positive predictive value (PPV) of 66%, and negative predictive value (NPV) of 95% [25]. However, it should be noted that the PRESEP study carries a significant risk of bias and, in fact, reports lower specificity (29%) and positive predictive value (41%) than previously indicated [26].

The literature presents a wide range of reported sensitivity and specificity values for the mRST in prehospital settings (Table 3). McClelland et al., report a sensitivity of 30% and specificity of 77% [28]. On the other hand, Wallgren et al. suggest a sensitivity of 93% without reporting the corresponding specificity [27]. Jouffroy et al., in their study, suggest a sensitivity of 100%, a specificity of 16%, a PPV of 39%, and a NPV of 100% with a threshold of ≥ 2 [26]. Lastly, Bayer et al. suggest a sensitivity of 95%, a specificity of 43%, a PPV of 32%, and an NPV of 97% [25].

The precision diagnostic of MEWS, similar to mRST, exhibits variation for prehospital diagnosis across different literature sources. For instance, Jouffroy et al. propose a sensitivity of 85%, specificity of 33%, PPV of 41%, and NPV of 88% when using a threshold of ≥ 5 [26]. Conversely, Bayer et al. propose a sensitivity of 74%, specificity of 75%, PPV of 45%, and NPV of 91% [25].

Hargreaves and colleagues conducted a study to assess the associations between the NEWS, lactate levels, and mortality in a large sample of patients with suspected sepsis in prehospital settings. The authors concluded that a persistent elevation of NEWS ≥ 5 from prehospital to ICU admission, along with an elevated lactate level of ≥ 2 mmol/L, can be used to identify patients at high risk of mortality in suspected sepsis cases [29]. However, the specificity and precision of this screening tool still require further investigation. It is important to note that SIRS, NEWS, and MEWS continue to serve as screening tools for sepsis or SS within the context of the Surviving Sepsis Campaign [14].

The SIGARC score (Table 4) was created by Jouffroy et al. as a screening tool specifically designed for identifying SS cases in prehospital settings when surpassing the threshold of ≥ 2, with a sensitivity of 76%, specificity of 41%, PPV of 38%, and NPV of 79% [12]. Additionally, the authors suggest that a SIGARC score of ≥ 2 is also associated with an increased risk of 30-day and 90-day mortality, with hazard ratios (HR) of 1.57 (95%CI 1.02–2.42) and 1.82 (95%CI 1.21–2.72), respectively [12].

The diagnosis of sepsis and SS in prehospital settings presents a significant challenge, as demonstrated by previous research. Dorsett proposes the existence of an occult state of sepsis and SS, wherein patients have not yet entered a critical disease trajectory; this state can potentially be identified through the use of screening tools at an early stage, which would result in the greatest benefit for the patient from early interventions [11]. Therefore, it is imperative to incorporate and utilize screening tools that take into account both macro and micro cellular perfusion markers, such as capillary refill time (CRT) and skin mottling score (SMS). Additionally, it is essential to integrate markers and surrogates of cellular metabolism, such as end-tidal carbon dioxide (EtCO2) and lactate. Previous studies have demonstrated a significant correlation between lactate levels greater than 2 mmol/L and CRT exceeding 3 s [30, 31]. Furthermore, an EtCO2 level below 25 mmHg has been identified as a valuable diagnostic tool for suggesting and diagnosing sepsis and SS, given its underlying pathophysiology; notably, a strong association has been observed between EtCO2 levels below 25 mmHg and lactate levels surpassing 4 mmol/L [30, 31]. However, it is important to exercise caution when utilizing EtCO2, as Weiss et al. have reported that low EtCO2 levels can predict initial lactate levels but do not accurately predict the diagnosis of infection; in their study only 27% of the total patients with low EtCO2 levels were found to have an infection at diagnosis [31].

Due to the variability in sensitivity, specificity, positive predictive value (PPV), and negative predictive value (NPV) among the reviewed screening tools, it is not possible to recommend a specific tool. Further studies in prehospital settings are needed to establish an appropriate screening tool for sepsis and SS for EMS use.

#### Prehospital management of septic shock

**Fig. 2:**
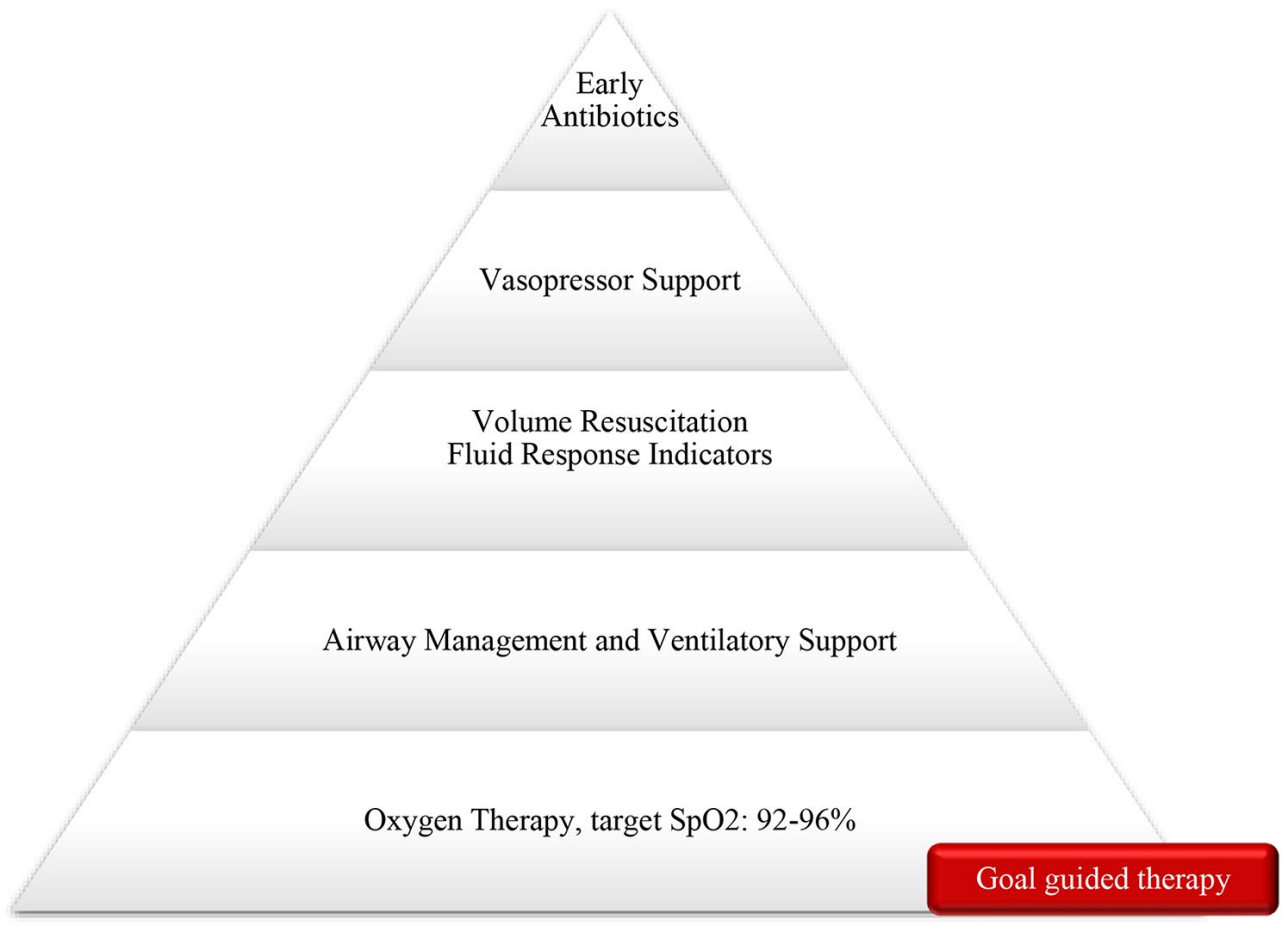
Septic shock prehospital management pyramid

The implementation of prompt and proactive interventions in the treatment of SS has been identified as a fundamental approach to enhance the likelihood of survival [13]. Given the complexity of SS, its pathophysiology, and clinical manifestations, EMS must adopt a comprehensive, evidence-based approach to management. This approach should focus on treating life-threatening conditions and improving survival rates. Key interventions in prehospital settings include oxygenation, ventilatory support, hemodynamic support, and early empiric antibiotic therapy [15, 17, 22, 32]. These interventions must be dynamic and individualized, incorporating knowledge of pathophysiology, patient comorbidities, clinical manifestations, disease state, available resources, and adherence to relevant policies and regulations (Fig. 2).

### Oxygenation

Oxygen therapy is a primary intervention utilized by EMS to mitigate the harmful effects of hypoxia. However, care must be employed as uncontrolled administration of oxygen, where a high fraction of inspired oxygen (FiO2) is maintained until the target peripheral oxygen saturation (SpO2) reaches 100%, has been associated with reduced survival rates and increased complications (due to the release of free radicals, which can exacerbate the dysregulated inflammatory response observed in SS) [32, 33].

Even short periods of hyperoxia during prehospital support have been directly correlated with increased mortality [32]. During prehospital care Jouffroy et al. found a 57% mortality rate when arterial partial pressure of oxygen (PaO2) exceeded 150 mmHg, compared to 18% when PaO2 was below 100 mmHg [32]. Therefore, healthcare providers are advised to maintain normoxemia (SpO2 between 92 and 96%) and accept relative hypoxemia (SpO2 between 88 and 92%) in patients with chronic hypercapnia [33].

### Airway management and ventilatory support

Basic and advanced procedures should be used to maintain airway patency and ventilatory support, depending on the patient’s condition, resources, and healthcare professional skill [34]. A complete clinical evaluation should determine advanced airway treatment based on the patient’s state of consciousness, respiratory rate, breathing effort, hemodynamic status, and capnography and oximetry gas exchange assessment [34].

SS patients’ induction agent choice is important during rapid sequence intubation (RSI) or rapid sequence airway (RSA). Unfortunately, our scoping investigation found no prehospital information on this topic, and hospital material is inconsistent.

Etomidate can stabilize hemodynamics in hypotensive individuals, although it may promote adrenal insufficiency and increase SS mortality [35]; however, current research does not link etomidate to adrenal insufficiency in septic individuals [35, 36]. In contrast, ketamine is effective for SS induction and sedation. Ketamine exhibited a better hemodynamic profile than fentanyl for anesthesia induction in SS patients, with a reduced frequency of hypotension (47.8%) compared to 84.2% [37]. A research comparing ketamine and etomidate in septic RSI patients reported no significant hemodynamic response or mortality differences [35]. The combination of half-dose ketamine and lidocaine during RSI reduced hypotension and improved mean arterial pressure (MAP) [38]. Ketamine is being used as the principal induction agent in RSI in SS patients, however prehospital data is scarce. Therefore, more research is needed to determine ketamine’s safety and efficacy in these situations.

In comprehensive septic shock (SS) care, airway patency and a secure seal between the airway device and the patient’s airway are critical for monitoring lung pressures and regulating gas exchange [26]. Due to its simplicity and high success rates, supraglottic airway devices may be preferred over orotracheal tubes in prehospital care (particularly in non-physician-staffed services) [39]. These devices do not seal the airway like endotracheal tubes. In addition, prehospital intubation success rates range from 46 to 95%, with aeromedical personnel often having the highest rates [15, 39, 40]. This unpredictability emphasizes the need for trained clinicians, especially for SS patients in the prehospital setting.

Finally, when requiring mechanical ventilation, the implementation of a lung protective strategy involving the use of low tidal volume has demonstrated enhanced survival rates in SS patients prior to their admission to the intensive care unit. Specifically, a tidal volume indexed on ideal body weight (VTIBW) of less than 8 ml/kg was found to be associated with a reduction in mortality, whereas a VTIBW exceeding 8 ml/kg was linked to an increase in mortality within a 28-day timeframe [15].

### Hemodynamic support

The primary objective of hemodynamic support is to restore adequate tissue perfusion pressure. The recommended perfusion pressure is a mean arterial pressure (MAP) of at least 65 mmHg; however, research indicates that patients with chronic hypertension may require a higher threshold, with a MAP of at least 75 mmHg even in prehospital settings [22]. To optimize patient outcomes, blood pressure management should be tailored to the specific needs of each individual. This involves assessing the perfusion state of various bodily systems, including cutaneous, renal, and neurologic systems, and using capillary refill time as a proxy for lactate level [7, 41].

The implementation of prehospital hemodynamic optimization has demonstrated a reduction in mortality rates among patients with SS. Adequate stabilization of hemodynamic parameters has been found to be correlated with a decrease in 30-day mortality (HRa = 0.52, 95% CI [0.31–0.86], *p* = 0.01) [22]. This process involves the administration of fluids to increase volume within the initial hours, followed by the use of vasoactive amines if the MAP remains below optimal levels [22].

### Fluid response indicators

The 2021 Surviving Sepsis Campaign Guidelines (SSC) advocate fluid delivery at 30 ml/kg within 3 h for septic patients, while other writers disagree [14]. Overloading with fluids worsens clinical conditions; therefore, patient response tests such passive leg raises (PLR), plethysmography variability index (PVI), fluid challenges, or bedside ultrasonography –with point-of-care ultrasound (POCUS)– investigations should be used to determine fluid needs [14, 18]. These tests are useful in prehospital settings, but in-hospital trials provide the most data. Unfortunately, our scoping analysis could not find any information on prehospital fluid response markers, thus more research is needed to support their use. In-hospital fluid response markers should perform similarly in the pre-hospital scenario, thus we included them in our review.

A positive response to fluid administration is defined as a stroke volume index rise of more than 10% or a perfusion index increase of more than 9% (sensitivity of 91% and specificity of 79%) for PLR; a PVI greater than 14; or an inferior vena cava diameter variation of more than 50% during inspiration in POCUS (a variation of less than 12% indicates non-responsiveness) [42–46]. A combination of these techniques can help in reducing fluid delivery, starting vasopressor support earlier, enhancing perfusion and oxygenation, and preventing pulmonary edema [45]. However, a comprehensive meta-analysis found that volume-based resuscitation did not reduce sepsis mortality overall [46]; therefore, more evidence is needed.

### Fluid therapy

Hemodynamic resuscitation begins with fluid volume expansion. Academics disagree on the ideal volume, especially in prehospital environments with little data. A 2018 retrospective study examined fluid delivery related to optimum body weight and prehospital SS mortality [47]. This study demonstrated a significant connection between mortality and fluid expansion when indexed on ideal body weight less than 10 ml/kg (OR = 4.17 [95%CI: 1.89–9.43]) or larger than 20 ml/kg (OR = 0.27 [95%CI: 0.12–0.57]), but not between 10 and 20 [47].

A retrospective study by Jouffroy et al. in 2021 examined the relationship between fluid administration, hemodynamic optimization (MAP > 65 mmHg or > 75 mmHg in hypertension patients), and prehospital SS mortality. In the first hour, they gave 15 ml/kg/hour, half the recommended 30 ml/kg in three hours. They found no variation in fluid expansion indexed on real and ideal body weight. Patients who achieved hemodynamic optimization received a median volume of 800 ml (range: 500–1250 ml) compared to 750 ml (range: 500–1000 ml) in those who did not, which reduced mortality [47]. A recent multicenter trial of 60 US centers found no significant difference in mortality between liberal and restrictive fluid strategies within 24 h [48]. Some suggest that moderate to high-volume fluid resuscitation (15–20 ml/kg) may be optimal [22, 47].

We cannot recommend a precise volume of fluids for prehospital fluid resuscitation due to a lack of data. Larger prospective trials are needed to understand the fluid volume expansion threshold and how prehospital hemodynamic adjustment reduces SS mortality.

### Vasopressor support

Vasopressors were often delayed in the prehospital setting due to concerns about their safety when given through a peripheral intravenous catheter. Recently published systematic evaluations have found peripheral intravenous catheters safe for 24 h of vasopressor therapy [49, 50]. The prehospital administration of vasopressors reduces SS mortality. In a retrospective review of 478 SS patients receiving prehospital vasopressors, specifically norepinephrine to attain a goal MAP > 65 mmHg, 30-day mortality was reduced by 0.42 (95%CI: 0.25–0.70) [51].

Safety and efficacy make norepinephrine the field’s favored vasopressor [52, 53]. Epinephrine is an effective secondary option, while dopamine is avoided [52, 53]. In life-threatening hypotension, push-doses of epinephrine or norepinephrine might be used until a continuous infusion is established [52, 53].

Guidelines recommend starting vasopressor therapy immediately, although the appropriate threshold is unknown, especially in prehospital settings with little data. Recent proposals include the diastolic shock index (DSI = HR/DBP) to determine severity and suggest early vasopressor administration. A DSI > 2 implies severe shock and vasopressor use [54]. More study is needed to prove its usefulness in hospital and prehospital settings.

### Antibiotic therapy

International standards recommend administering broad-spectrum antibiotics within an hour after SS diagnosis to increase survival [14]. Despite reliable prehospital SS diagnosis, admission and antibiotic initiation times vary. Using a direct admittance system from the prehospital service, Peltan et al. found that antibiotic therapy was started earlier − 103 min after medical diagnosis [16]. According to Seymour et al., for each hour of delay in antibiotic therapy, the OR for mortality increases by 1.04 (95% CI: 1.03–1.06) [21]. In SS patients, Im et al. found that every hour of antibiotic delay increases mortality by 35% [55].

Given the delays in hospital antibiotic initiation, prehospital antibiotic administration may improve survival. A 2022 systematic review and meta-analysis by Varney et al. found that antibiotics before hospitalization can significantly reduce sepsis and SS mortality. Prehospital antibiotics reduced sepsis and SS mortality risk by 0.81 (95% CI: 0.68–0.97, *p* = 0.02) [56]. Paramedics should also take blood cultures before giving antibiotics [57]. Antibiotics work in prehospital settings, but they should not be used carelessly. Prehospital clinicians must be trained to reliably identify sepsis, use suitable detection methods, run blood cultures, and deliver intravenous antibiotics according to medical recommendations to guarantee safe and effective administration.

## Conclusions

SS is a critical condition that poses a significant risk to life. Early and accurate identification of SS, as well as prompt initiation of appropriate treatment in prehospital settings, are considered crucial factors that can greatly enhance the likelihood of survival. The diagnosis of SS poses a significant obstacle in prehospital environments, necessitating a comprehensive understanding of pathophysiology, clinical manifestations, and the utilization of various indicators of cellular perfusion and metabolism that are accessible in these settings. The present approach to managing SS involves addressing oxygenation, providing ventilatory support, offering hemodynamic support, initiating early antibiotic therapy, and prioritizing these interventions as key strategies to reduce mortality in patients with SS. However, additional studies and randomized clinical trials are necessary to improve prehospital guidelines for SS in the future.

## Data Availability

All data generated or analyzed during this study are included in this published article.
